# DOACs in the Anticoagulation of Mechanical Valves: A Systematic Review and Future Perspectives

**DOI:** 10.3390/jcm12154984

**Published:** 2023-07-28

**Authors:** Tom Langenaeken, Arnaud Vanoppen, Fien Janssens, Loes Tanghe, Peter Verbrugghe, Filip Rega, Bart Meuris

**Affiliations:** Department of Cardiac Surgery, University Hospitals Leuven, Herestraat 49, 3000 Leuven, Belgiumbart.meuris@uzleuven.be (B.M.)

**Keywords:** DOAC, mechanical valve, anticoagulation, animal trials, human trials, mechanical valve thrombosis

## Abstract

Valvular heart disease is a common disease often necessitating valve replacement. Mechanical heart valves (MHVs) are often used in younger patients because of their longer durability. Their main disadvantage is the need for lifelong anticoagulation. Warfarin is considered a standard treatment, but it is far from perfect. Direct oral anticoagulants (DOACs) are a new and more patient-friendly alternative to warfarin when anticoagulation is required, but have not yet been approved for the indication of mechanical valves. Evidence acquisition: A literature search of Pubmed, Embase, Web of Science (Core Collection), and Cochrane Library (from inception to May 2023) was performed using a search string that was well defined and not modified during the study. An extensive overview of the search terms used in each database can be found in the Appendix. Only prospective clinical trials were included in this review. A total of 10 publications were included in this review. Relevance to clinical practice: This systematic review summarizes the different types of DOACs and their possible use in the anticoagulation of mechanical valves. We aim to propose future directions in anticoagulation research for mechanical valves. Conclusions: DOAC use in MHVs has been halted due to the failure of both dabigatran and apixaban in two major clinical trials. However, rivaroxaban was successful in two small clinical trials. Ample research is still needed to explore new valve designs as well as new anticoagulation targets.

## 1. Introduction

Valvular heart disease is currently affecting over 100 million people, increasing each year with the aging population [1]. Rheumatic heart disease is most prevalent in the young, while the elderly suffer from calcific aortic and degenerative mitral valve disease. If valvular heart disease progresses untreated, morbidity and mortality soon follow [2]. Valve replacement is the second most common major heart operation in the Western world. It is estimated that four million valve replacement procedures have been performed over the last 50 years [3]. Valve replacement remains the only definitive treatment for most patients with advanced heart valve disease [4]. In younger adults, the surgeon’s decision as to which valve is best can be difficult. Usually, the choice is made between a mechanical valve, a bioprosthetic valve, and the Ross procedure. The use of the Ross procedure remains limited, and the operative mortality is directly related to the surgeon’s experience and expertise [5]. In the other two options, the surgeon needs to balance the risk of thromboembolic and bleeding events mainly associated with mechanical valves (MHVs) and the need for long-term anticoagulation, against the risk of reoperation secondary to structural valve deterioration using bioprosthetic valves [6]. Mechanical valves have a longer durability, making them interesting for younger people. Their main disadvantage remains their thrombogenicity due to contact with the passing blood elements combined with a non-natural flow over the bileaflet valve [7,8].

To this day, lifelong anticoagulant therapy using vitamin-K antagonists (VKAs) remains necessary to avoid mechanical valve thrombosis. Warfarin has several shortcomings: a long half-life, inter- and intraindividual variations in response, genetic polymorphism alterations in warfarin metabolism and multiple food and drug interactions, and the inherent risk of bleeding complications [6,9,10]. Warfarin requires frequent monitoring and targeting of the international normalized ratio (INR) through serial blood sampling. We should not underestimate the impact on the quality of life in patients who have to be checked periodically for INR for the remainder of their life. There are self-monitoring programs in which the INR can be determined at home by using portable coagulometers [11]. This is a significant improvement in quality of life, yet it remains time-consuming and there is a risk of decreased therapy compliance [12]. The many disadvantages combined with the lifelong need for INR monitoring have driven the search for better alternatives to warfarin and its analogs [13,14].

DOACs proved to be a valid alternative to warfarin in various indications [15]. The advantages of DOACs include high bioavailability, rapid onset of action, wide therapeutic window, no food interactions, few drug interactions, predictable pharmacokinetic and pharmacodynamic profiles, and no coagulation monitoring [10].

In this systematic review, we summarize all the evidence regarding mechanical valve anticoagulation with DOACs in the (pre)clinical setting.

## 2. Materials and Methods

This systematic review is intended to summarize the most recent data on the topics “DOAC” and “heart valves”. It gives an overview of the different types of DOACs, current indications, and future directions. Our focus is on the use of DOAC as anticoagulation therapy in (pre)clinical trials (only in vivo experiments or human trials).

We searched all possible databases: Pubmed, Embase, Web of Science (Core Collection), and Cochrane Library (from inception to December 2022) with no language limitations, using the following search string: “Factor Xa Inhibitors” OR “Direct acting oral anticoagulant*” OR “novel oral anticoagulant*” OR “DOAC” OR “NOAC” OR “rivaroxaban” OR “apixaban” OR “dabigatran” OR “edoxaban” OR “factor 10a inhibitor*” AND “Heart Valve Prosthesis” OR “valve prostheses*” OR “valve replacement” OR “valve implantation”. This systematic review was conducted according to the Preferred Reporting Items for Systematic Reviews and Meta-Analyses (PRISMA) statement [16].

## 3. Results

**Figure 1:**
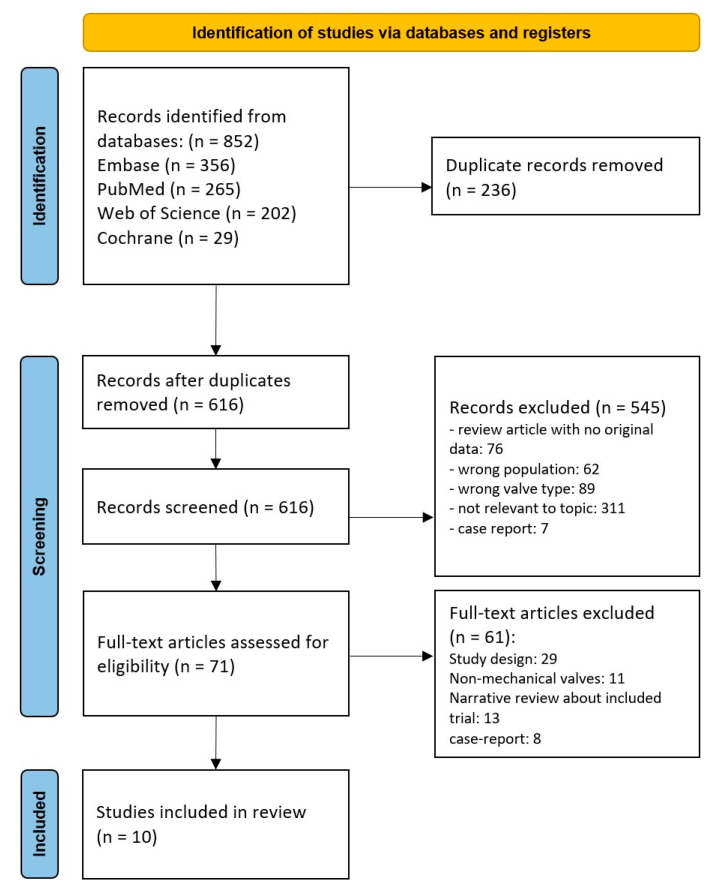
PRISMA flow diagram.

Our search strategy generated a total of 852 results from all the different databases listed above. After removing the duplicates using Endnote, 616 remained. We exported these articles to Rayyan, in which two people independently screened the studies by title and abstract [17]. A total of 71 articles were selected for full-text eligibility, from which 10 articles finally remained to be included in our review (see Figure 1).

Out of our 10 articles, 5 were animal models incorporating swine and 5 were human trials; 3 studies were found for dabigatran, 4 for rivaroxaban, and 3 for apixaban. No studies incorporating MHVs and edoxaban were found. All of these studies are summarized in Table 1, Table 2 and Table 3.

### 3.1. Mechanism of Thrombosis in Mechanical Heart Valves

Thrombus formation on mechanical valves is due to the activation of both intrinsic and extrinsic coagulation pathways. The artificial material in contact with the passing blood elements activates the intrinsic pathway through contact activation. The extrinsic pathway is activated by the shear stresses generated due to the non-physiological flow over the valve [7,8].

#### 3.1.1. Surface-Related Factors

Presumably, rapid adsorption of plasma proteins on the artificial surfaces is the initial stimulus of thrombus formation [26]. This rapid adsorption of fibrinogen and other proteins is facilitated by negatively charged hydrophilic surfaces independent of flow and leads not only to platelet adhesion but also to leukocyte and passive red blood cell adhesion. Activated platelets release thromboxane A_2_, adenosine diphosphate (ADP), and other agonists, further amplifying adhesion, activation, and aggregation [26]. Later, fibrinogen is replaced with other proteins of the contact system, such as factor(f) XII, high molecular kininogen (HK), prekallikrein, and fXI in what is known as the Vroman effect, meaning that surface adsorption is a reversible process with changes in the composition of absorbed proteins over time [26,27]. The activated fXII (fXIIa) ensures complement activation and generation of thrombin by stimulating this intrinsic pathway [28]. Consequently, due to the interaction between the complement and coagulation pathways, thrombin formation is further enhanced [29]. Moreover, fXIIa activates fXI, which will also lead to thrombin production. Thrombin then both converts fibrinogen to fibrin monomers and further enhances local platelet aggregation, which was already triggered by fibrinogen. After polymerization of those fibrin monomers, the fibrin strands form a platelet–fibrin thrombus. About 3 months later, this fibrin coat is replaced by a neointimal layer that becomes more fibrotic [17,30,31].

#### 3.1.2. Hemodynamic Factors

A distinction can be made between the intrinsic hemodynamic features of the valve and the hemodynamic status of the patient.

In the proximity of the mechanical heart valves, non-physiological blood flow patterns occur and contribute to thrombus formation [32]. When the valve opens, high turbulent shear stresses cause mixing of the blood composition which make it easier for blood platelets to come into contact with the endothelium and to form platelet-rich clots [31]. Moreover, high shear stress will also occur in the hinged gaps when the valve is closed [8]. Through these hinged gaps, jets are flowing backward into the heart with excessive shear stresses causing damage to the blood cells and platelets. This results in tissue factor release and thus activation of the extrinsic coagulation pathway [7,33].

Besides turbulence and shear stresses, certain locations of stasis can arise in and around mechanical valves (including the hinges and the wakes of the leaflets), especially when the valve is mispositioned [32,34]. Blood stasis increases blood coagulability by reducing washout and dilution of the clotting factors and limiting the inflow of anti-coagulation inhibitors at the same time. As a result, fibrin-rich clots can be formed [31]. Turbulence may also play a role in neointimal injury or dysfunction and delayed endothelization, leading to further activation of hemostatic mechanisms [29,34].

In addition, the localization of the valves has a role in thrombogenicity. Valve thrombosis is 20-fold more likely to develop in the tricuspid position than in the mitral position, but it is also 2-fold more frequent in the mitral valve than in the aortic valve [29,35].

Furthermore, the hemodynamic status of the patient can also favor thrombosis, specifically in states of low cardiac output or low flow states [35].

#### 3.1.3. Hemostasis-Related Factors

During the operation, local tissue injury and hemolysis can release tissue factor (TF) and thereby activate the extrinsic coagulation pathway: TF forms a complex with fVIIa that converts fX to fXa. Factor Xa provokes the generation of thrombin, which is subsequently augmented by the intrinsic factor, and eventually, a fibrin clot is formed [28,31]. This mechanism may be accountable for short- and mid-term local thrombogenicity [34].

Finally, primary or secondary hypercoagulable states may also contribute to the pathogenesis of valve thrombosis [29,34]. Some examples are FV Leiden, protein C or S deficiencies, atrial fibrillation, antiphospholipid antibody syndrome, pregnancy, smoking, and obesity. In all such conditions, just as in states with inadequate anticoagulation or recurrent thrombosis, the risk of thrombosis is likely to increase [35,36,37].

### 3.2. Mechanical Heart Valve Anticoagulation with DOACs: Animal and Human Studies

Several studies have attempted the anticoagulation of mechanical valves in animal or human trials. Dabigatran is the most well-known due to its earliest development and use in the RE-ALIGN trial [20]. After dabigatran, attention shifted to factor Xa inhibitors such as apixaban, rivaroxaban, and edoxaban.

#### 3.2.1. Dabigatran

Dabigatran is the first approved DOAC and is a direct thrombin (factor IIa) inhibitor. Thrombin is an essential factor at the end of the clotting cascade, which ensures the conversion of fibrinogen to fibrin. Dabigatran binds to one of the two thrombin sites and thus can inactivate thrombin [10]. It is taken orally as a prodrug (dabigatran etexilate) and is rapidly converted by the liver to the active form [38]. Dabigatran is currently used in the prevention and treatment of thromboembolic events, usually in the setting of non-valvular atrial fibrillation. It showed a more favorable risk–benefit profile compared to warfarin [39,40].

A limited number of studies have been conducted for dabigatran in MHVs, with one large randomized clinical study (see Table 1). McKellar et al. were the first to implement dabigatran in an animal model of mechanical valve replacement [18]. Their heterotopic descending aorta bypass graft model, first described in 2007, allowed the testing of different drug regimens in swine [18,21,41,42]. When applying dabigatran to this model, the thrombus formation on the valve was significantly reduced when compared to no anticoagulation or subcutaneous enoxaparin. The fact that less platelet deposition and no bleeding events were observed in the dabigatran group ensured a promising foundation for future prospective clinical trials. One of the potential issues with this model is the fact that the mechanical valve is not in an orthotopic position. One might question whether the valve leaflets open and close properly in the non-pulsatile flow of the descending aorta.

The next animal trial using dabigatran was by Schomburg et al. [19]. In this study, the valve was positioned orthotopically in the mitral position in swine. The primary endpoint was animal mortality, and the designed study length was 90 days or 3 months. Only the dabigatran cohort had four full-term survivors, differing significantly from the “no-anticoagulation” control and the warfarin group, having no full-term survivors. In addition, hemopericardium was seen more often in the warfarin cohort (2/5) than in the dabigatran group (2/10).

This study demonstrated the presence of thrombi in all three groups [19]. In 2/5 animals in the warfarin group and 8/10 in the dabigatran group, clear thrombi were found; 2 thrombi in the dabigatran group were infectious in nature (endocarditis). Schomburg does state, however, that in the four long-term dabigatran survivors, smaller thrombi were seen in three of them and a larger one in the fourth. It is known that pigs in the immediate postoperative period are in a relatively hypercoagulable state [43]. Next to this, it is difficult to achieve adequate anticoagulation levels in pigs immediately postoperatively due to two reasons: (1) the animals still have hampered ingestion as they are recovering; and (2) the presence of gastric ulcers (as demonstrated in this study), that can be responsible for the lower effectiveness of dabigatran. In conclusion, Schomburg states that dabigatran increased survival and decreased bleeding complications in his swine model, but refrains from making a statement regarding the impact of dabigatran on pure thrombus formation [19].

A striking feature of both studies is that the dabigatran dosing given was up to 9.3 times higher than the normal human dose [29]. Of course, the dosing in these studies relies on current knowledge of animal models with respect to the pharmacokinetics and pharmacodynamics of DOACs in swine. It is, however, disturbing, that even at these high doses, valve thrombi are seen in a relatively short time span. This brings into question either the validity of these models or the efficacy of dabigatran in swine.

The final question as to whether dabigatran was capable of properly anticoagulating a mechanical valve was answered in the RE-ALIGN trial [20]. In this trial, 252 patients with either a mitral or aortic mechanical valve, or both, were recruited. Patients were either recruited immediately after surgery (*n* = 199) or >3 months post-surgery (*n* = 52). Randomization in a 2:1 ratio led to 84 patients in the warfarin group and 168 in the dabigatran group. Warfarin was adjusted according to INR (2–3 with no additional risk factor, 2.5–3.5 with an additional risk factor or mitral mechanical valve). Dabigatran dosing was according to kidney function, 150/220/300 mg bidaily (BID). Dabigatran trough levels were measured within the first 2 weeks of recruitment. If levels were lower than 50 ng/mL, patients were upgraded to the next higher dose.

Unfortunately, the trial ended prematurely due to an excess of both thromboembolic and bleeding events in the dabigatran group. After 140 days, nine patients (5%) in the dabigatran group and none in the warfarin group suffered a stroke. In addition, major bleeding occurred in the dabigatran group in seven patients (4%) vs. two patients (2%) in the warfarin group. The results of this landslide study practically completely halted research on this topic. However, many questions were raised after critical analysis.

An important factor that stands out is the difference in dosing. Concentrations used to achieve relative success in animal experiments (500 nmol/L) are several factors higher than those measured in the RE-ALIGN trial [44]. One would argue that the trough level of 50 ng/mL based on the results of the RE-LY study was too low [45]. However, even at this level, excess bleeding was already seen. Should one strive for the same blood levels at which success in vitro can be achieved (1000 nmol/L), the bleeding rates would be unacceptably high [46].

The increased incidence of stroke in the dabigatran arm could be explained by the difference in pharmacodynamics between dabigatran and warfarin. Warfarin is a strong anticoagulant with inhibition of the tissue-factor-pathway- (factor VII), contact-pathway- (factor IX), and common-pathway-induced coagulation (factors X and II, as well as protein C and S). The tissue factor pathway plays the most important role in the development of thrombosis in MHVs [47]. Dabigatran, on the other hand, only inhibits thrombin (factor IIa), making it less potent in patients with an MHV [47].

A second problem according to the RE-ALIGN study group is the increased incidence of bleeding in the dabigatran group. This could be explained by the pharmacokinetic effects of dabigatran. This drug has a low oral bioavailability of 6–7%, leaving a large portion of the drug in the lumen. Dabigatran as a prodrug is activated in the liver, yet this drug is also activated in the gut by unknown mechanisms [48]. This could explain the increased risk of gastrointestinal bleeding due to local action, as a possible explanation for the increased level of gastric ulcers seen in Schomburg’s swine experiment [19].

#### 3.2.2. Rivaroxaban

Due to the disappointing results of the factor IIa inhibitors, attention shifted to more upstream factors of the coagulation cascade. Rivaroxaban is an oral inhibitor of both free and prothrombinase complex factor Xa [21]. The ROCKET AF clinical trial showed that rivaroxaban is not inferior to warfarin in the prevention of stroke or systemic embolism in patients with atrial fibrillation [49]. It is also proven safe for the prevention of both arterial and venous thrombosis after orthopedic surgery and in the treatment of pulmonary embolism [50,51,52].

Only one important animal trial with rivaroxaban for mechanical valve anticoagulation has been conducted, by Greiten et al. [21]. Using the same heterotopic descending aorta valve conduit designed by McKellar et al., 30 swine were implanted with a St. Jude Masters mechanical valve in the descending aorta. Equal groups of 10 animals each were designated, receiving either no anticoagulation, enoxaparin, or rivaroxaban. At 30 days, significantly lower thrombus weight and platelet deposition were seen in the rivaroxaban group. These relatively encouraging results did not lead to more animal trials. The same remark about (the absence of) adequate and normal valve function in the descending aorta remains.

Two small human trials have been performed. Durães et al. were the first in 2018 [4]. A small selection of seven patients with difficulties in maintaining adequate INR after isolated mitral valve replacement were selected, at least 3 months postoperatively. Medication was switched to rivaroxaban 15 mg BID and follow-up was 3 months. Follow-up consisted of transesophageal echocardiography (TEE) to exclude subclinical valve thrombosis, and spontaneous echo contrast (SEC) or intracardiac thrombus and computed tomography (CT) head scans to exclude infarction or cerebral hemorrhage before and after rivaroxaban use. During the 3 months of follow-up, patients were contacted by phone weekly and a transthoracic echocardiogram (TTE) was performed every 30 days. After 3 months, no abnormalities of any kind could be noted. Spontaneous echo contrast even disappeared in two patients. The researchers even noted high patient satisfaction, with most patients willing to continue their rivaroxaban anticoagulation treatment.

Another human trial was conducted 2 years later by Roost et al. [22]. This study design differs in several key points: (1) patients were not selected based on previous poor experiences with warfarin; (2) patients were included immediately after surgery, in analog to the RE-ALIGN trial treatment group A; (3) the valve used was the Medtronic Open Pivot prosthesis in the aortic position. The open pivot system is unique due to the absence of cavities or recesses where thrombi may form [53]. It claims to provide passive washing of the hinges by unhampered blood flow with minimal hemolysis, reducing thrombosis risk [54]. The 10 patients included received rivaroxaban 20 mg OD, which is significantly less than the 15 mg BID dosing in the Durães et al. trial [4]. The follow-up was twice as long, being 6 months in total. Follow-up consisted of TTE and transcranial Doppler at discharge. At 90 and 180 days, a full neurological examination combined with laboratory testing, TTE, and transcranial Doppler was performed. At the end of follow-up, no abnormalities of any kind were noted. There were no neurological abnormalities of any kind. Echocardiographic parameters did not differ from those at hospital discharge and valve function was completely normal. Laboratory testing was also completely normal.

Building upon their 2018 pilot trial, Durães et al. included 44 patients randomized 1:1 to receive either dose-adjusted warfarin or rivaroxaban 15 mg twice daily [23]. Follow-up was extensive and comparable to their pilot study 2 years earlier. The duration of the study was 90 days and patients were at least 3 months post-operation when rivaroxaban was started. One major difference with the pilot study was that aortic, mitral, and double valve procedures were eligible for study participation. After 90 days, one patient in the rivaroxaban group experienced a transient ischemic attack (TIA) and six reported minor bleeding (without discontinuation of medical therapy). In the warfarin group, ischemic stroke occurred in two patients and silent brain injury (SBI) in one. Also in the warfarin group, one patient died from an acute myocardial infarction, and minor bleeding occurred in six patients. There were no clinical signs of valve thrombosis of any kind. The researchers concluded that at 90 days, no statistical difference in any outcome between rivaroxaban and warfarin was assessed. Numerically, however, rivaroxaban had fewer events.

These four studies make a case for the use of rivaroxaban in the setting of mechanical valve anticoagulation. Especially the study by Roost et al., where a relatively low dose of only 20 mg once daily (OD) was used, gives hope for a future of DOAC use in the setting of mechanical valve anticoagulation. Several explanations for their success are given when compared to the failure of the RE-ALIGN trial. First, only isolated aortic valve replacements were included, in contrast to Eikelboom et al., who also included double valve replacements. Roost et al. also state that compliance with a once-daily drug intake compared to a twice-daily intake might be higher in their study. The main limitation is obviously their small sample size and limited follow-up, narrowing the time and statistic window to detect any adverse events. However, the RIWA trial by Durães et al. does include aortic, mitral, and double valve replacements parallel to the RE-ALIGN trial [20,23], although the follow-up period is shorter. Another important difference between the RIWA and RE-ALIGN trials is that the concomitant use of antiplatelet medication was prohibited to avoid any confounding.

The combined results of these four studies allow us to state that a factor Xa inhibitor proves a better target than factor IIa inhibitors. Rivaroxaban has an oral bioavailability of 80%, compared to 7% for dabigatran [55]. As for dabigatran, the results of the RE-ALIGN trial halted further exploration of rivaroxaban in the setting of MHV anticoagulation.

#### 3.2.3. Apixaban

Apixaban is a DOAC that, like rivaroxaban and edoxaban, inhibits both free and clot-bound factor Xa [56]. Factor Xa serves as a medium between both intrinsic and extrinsic coagulation pathways, both of which are activated by MHVs [57]. Several indications have already been approved, including the prevention of venous thromboembolic events (VTE) in elective hip or knee replacement surgery, treatment and prevention of deep vein thrombosis (DVT) and pulmonary embolism (PE), and prevention of stroke and systemic embolism in non-valvular atrial fibrillation (NVAF) [58].

The first preclinical study was performed on swine by Lester et al. in 2017 [6]. In this study, sixteen swine received a descending aorta heterotopic mechanical valve replacement and were allocated into four groups (Table 3). The main outcome was that apixaban was equally effective in preventing thrombus formation in MHVs, demonstrated by a significant difference in thrombus weight between the treated groups and placebo. Apixaban PO had a slightly higher thrombus weight than warfarin: 357.5 ± 234.9 mg vs. 247.1 ± 134.3 mg, respectively, although this was not significant. Apixaban IV scored the best with the lowest thrombus weight of 61.1 ± 47.2 mg; however, it is important to note that these swine were sacrificed on day 14, as opposed to day 30 in the other groups. Another important aspect of this study was that bleeding events happened only in the warfarin group in two of the three swine, while no bleeding events were observed in the nine swine treated with apixaban.

Five years later, in 2022, another preclinical study evaluating apixaban in MHVs was published by Van Hoof et al. [24]. This research team placed mechanical valves in the highly thrombogenic pulmonary position [59]. Three groups were created out of nine swine: low-dose (2 × 5 mg), intermediate-dose (2 × 5 mg for 6 weeks followed by 2 × 10 mg for 4 weeks), and high-dose (2 × 15 mg) groups. The follow-up time was 10 weeks. Although thrombus weight did not differ significantly between groups, there was a clear negative correlation between apixaban dose and thrombus weight or evidence of clot. After ten weeks, evidence of clot was found in 2/2 (100%) in the low-dose, 2/4 (50%) in the intermediary-dose, and 0/3 (0%) in the high-dose group. No bleeding events were observed in this trial, indicating that increasing doses of apixaban added no additional bleeding risk.

Although these studies did not intend to explore the safety profile of apixaban, bleeding events did not occur when treated with apixaban. Previous studies have already shown that apixaban has the lowest bleeding risk of all the DOACs [60,61]. Furthermore, these two studies show that apixaban has positive results in the reduction and prevention of thrombus formation on MHVs. Of course, the evidence remains limited due to the low power of both studies and the absence of further follow-up research.

In addition, it is important to note that apixaban has a bioavailability of ± 50% in humans, which is greater than the 32% measured in the swine of these studies. Furthermore, the half-life in humans is ± 12 h compared to 1.6 h in swine [6]. One could imagine that positive results in swine should be translatable to even better results in humans.

This question was answered in the form of the PROACT-Xa trial, comparing the efficacy and safety of apixaban in the setting of mechanical aortic valve replacement compared to standard warfarin [62,63]. The trial was designed with the drawbacks of the RE-ALIGN trial in mind: one valve type was selected (the On-X valve), patients were only included at least 3 months postoperatively, only single aortic valve replacement patients were included, and most patients were on concomitant aspirin [29]. The primary efficacy endpoints were valve thrombosis or valve-related thromboembolism comparing apixaban with warfarin for noninferiority, and with an objective performance criterion (OPC) for mechanical valves set by the FDA [63,64].

In total, 863 patients were randomized: 430 were allocated to apixaban and 430 to warfarin. In the apixaban cohort, 3 valve thrombosis and 17 valve-related thromboembolic events were noted leading to an event rate of 4.2%/patient-years. Compared to the absence of valve thrombosis and only six valve-related thromboembolic events in the warfarin group, noninferiority for apixaban could not be met and the OPC for valve thrombosis or valve-related thromboembolism (3.4%/patient-years) was exceeded. The trial was stopped prematurely after less than 2 years [25]. With regard to bleeding events, there were 17 major bleeding events in the apixaban cohort and 18 in the warfarin group. These rates were not significantly different.

Another striking difference with the RE-ALIGN trial was the fact that most patients had concomitant aspirin. At randomization, 94.2% in the apixaban cohort and 94% in the warfarin cohort were on aspirin. This dropped to 84.5% in the apixaban cohort and 84% in the warfarin group for the remainder of the study, but still remained relatively high. The warfarin INR target was 2.0–3.0 as this is the most common standard, despite the fact that On-X valves are approved for a lower INR target of 1.0–2.0 [65].

Thus, despite the best trial design and investigator efforts, apixaban as the best possible currently available DOAC could not succeed in properly anticoagulating a recent mechanical valve in the least thrombogenic aortic position. This marks the second large trial failing in the anticoagulation of mechanical valves with DOACs, and it may very well be the last major trial in this direction. However, one new clinical trial was registered in January 2023. The Direct Oral Anticoagulation and Mechanical Aortic Valve (DIAMOND) trial will analyze the non-inferiority of apixaban 5 mg twice daily vs. standard warfarin 7 days after aortic mechanical valve replacement [66]. At the time of writing, recruitment had not yet started. However, with the poor results of the PROACT-Xa trial, one could question the usefulness of this trial.

#### 3.2.4. Edoxaban

Edoxaban was approved by the FDA in 2015 for the treatment of DVT, PE, and the reduction in the risks of stroke and systemic embolism in patients with nonvalvular AF [57]. Edoxaban inactivates clot-bound factor Xa. Edoxaban is however associated with gastrointestinal (GI) bleeding (like dabigatran), leading physicians to refrain from extensive use.

With our search strategy, we were unable to find any articles on edoxaban as an anticoagulant for thrombus prevention in MHVs at the time of writing. No clinical or preclinical (animal, or in vitro) studies have been conducted. Preclinical models evaluating its efficacy in the case of mechanical valve anticoagulation are warranted to further elucidate the true potential of this DOAC.

## 4. Discussion

The need for a valid alternative to VKAs in the setting of MHV anticoagulation remains unmet. DOACs are indispensable in the current practice. Research progress on this subject was largely halted after the RE-ALIGN trial in 2013. The decreasing numbers of mechanical valves implanted yearly combined with the increase in durability of biological valves and alternatives such as the Ross procedure have made it less opportune for the industry to invest in the development and testing of DOACs in the setting of MHV anticoagulation. This, combined with the recent failure of the PROACT-Xa trial, complicates future research in this direction.

One should refrain from using factor IIa inhibitors such as dabigatran, as this is too far downstream of the coagulation cascade. Clotting on MHVs generates massive amounts of thrombin by the contact activation pathway. Each molecule of factor Xa activates 1000 molecules of thrombin [47]. This was key to the failure of the RE-ALIGN trial: the dabigatran present in the blood was not nearly sufficient to adequately suppress the constant thrombin generation by a mechanical valve. Doses up to 620 mg BID would be needed, more than double the maximum dose administered in the trial [20,29,47].

Central to the hypothesis that factor X inhibition is sufficient for mechanical valve anticoagulation is the bridging of VKAs by unfractionated heparin (UFH) or low-molecular-weight heparin (LMWH) in the setting of pregnancy or upcoming surgery. In a meta-analysis of 1068 patients in 10 different studies, the temporary use of LMWH had similar outcomes to UFH or VKAs with regard to thromboembolic risk or bleeding events [67]. Similar safety has also been observed in atrial fibrillation patients temporarily bridged with LMWH [67]. However, the failure of the PROACT-Xa trial, where the dosing was identical for virtually all patients, brings into question the potency of factor Xa inhibition. The excess of thromboembolic events in the apixaban cohort could not be attributed to recent surgery, inappropriate dosing, interruption of apixaban, or any other identifiable factor [63].

Durães et al. and Roost et al. performed the only human trials where the efficacy of a DOAC in MHV anticoagulation was shown [22,23]. Hence, the DOAC meriting a place in future clinical trials should be rivaroxaban, ideally in a 15 mg BID dosing scheme [22,23]. An ideal patient would be recruited more than 3 months postoperatively after isolated aortic valve replacement. It is well-known that thrombosis risk is elevated in the immediate postoperative period, even in patients with bioprostheses [68]. The valve itself would need to be a recent valve, for example the On-X valve, which was approved for a lower INR reference range [65]. Although other valves, such as the Bicarbon valve, have quite similar data [69]. Preserved systolic function, low bleeding risk, no hypercoagulability, and good therapy compliance would be key factors to success [29].

Current developments in new types of DOACs merit our attention. Key in device-related thrombosis is protein absorption and FXII activation in the contact phase activation [26]. Anti-FXIIa antibody reduced clotting to the same extent as heparin in a rabbit model of extracorporeal membrane oxygenation without an increase in bleeding [70]. This follows the rationale that the higher the inhibition in the coagulation cascade, the better the outcome due to the amplification of coagulation factors downstream. One should intervene in the coagulation cascade before the formation of thrombin is imminent, as thrombin not only activates fibrinogen to fibrin monomers but also promotes local platelet adhesion [26]. Once these positive feedback mechanisms are active, inhibition of factor Xa or factor IIa in a 1:1 fashion, as in the RE-ALIGN trial and PROACT-Xa, is too late.

Newer bileaflet mechanical valves allow for lower INR ranges due to better materials used and optimal flow over the valve. The only bileaflet mechanical valve currently approved for a lower INR range (1.5–2.0) is the On-X valve by Cryolife [65]. It achieves this through a number of features, such as its pure pyrolytic carbon design, a flared inlet, increased height-to-cylinder ratio, 180° leaflet opening, and increased ‘washing’ of potentially thrombogenic hinge points [65]. Despite the failure of the On-X with apixaban in the PROACT-Xa trial, the combination of rivaroxaban and the On-X valve may still be successful. Trileaflet designs such as the Triflo valve by Novostia are also of interest [71]. The Triflo valve aims to be the first mechanical valve that is truly not thrombogenic due to minimal flow disturbance over the valve combined with minimal shear stresses [72]. Clinical first-in-man trials are currently planned after successful animal trials.

## 5. Conclusions

This systematic review provides the latest data regarding preclinical in vivo and clinical studies analyzing the efficacy of MHV anticoagulation using DOACs. Adequate animal models that allow good translation to clinical practice, as well as further elucidation of new bileaflet or trileaflet designs and insights into FXIIa inhibition are warranted. Clinical trials should focus on adequate patient and DOAC selection combined with intensive follow-up starting at least 3 months postoperatively. 

## Figures and Tables

**Table 1:** Dabigatran for mechanical valve anticoagulation.

Reference	S. McKellar et al. (2011) [18]	J. Schomburg et al. (2012) [19]	J.W. Eikelboom et al. (2013) [20]
Setting	Swine heterotopic aorta descendens valve conduit	Swine mitral valve replacement	Human aortic- or mitral-valve replacement: -within the past 7 days -replacement at least 3 months earlier
Treatment groups	10 swine: dabigatran 10 swine: enoxaparin 10 swine: no anticoagulation	3 swine: no anticoagulation 5 swine: warfarin 11 swine: dabigatran	84 patients: warfarin 168 patients: dabigatran
Dose	Enoxaparin 2 mg/kg BID SC Dabigatran 20 mg/kg BID PO	Warfarin INR 2–2.5 PO Dabigatran 20 mg/kg BID PO	Warfarin INR 2–3/2.5–3.5 * PO Dabigatran 20 mg/kg BID PO **
Primary endpoint	Amount of valve thrombus at 30 days.	Animal survival at 90 days.	Trough plasma level of dabigatran
Results	1 premature death in no anticoagulation group due to sepsis	Survival: -No anticoagulation: 18.7 days -Warfarin: 15.6 days -Dabigatran: 50.3 days	Terminated prematurely due to excess thromboembolic and bleeding events in the dabigatran group
Valve thrombus	No anticoagulation: 638 ± 895 mg Enoxaparin: 121 ± 128 mg Dabigatran: 19 ± 31 mg	2/3 control group 2/5 warfarin group 8/10 dabigatran group	N/A
Bleeding	None observed	2/5 warfarin group (hemopericardium) 2/10 dabigatran group	Any: dabigatran 27% vs. 12% warfarin (HR: 2.45 (1.23–4.86)) Major: dabigatran 4% vs. 2% warfarin (HR: 1.76 (0.36–8.46))
Stroke	N/A	N/A	Dabigatran: 9 patients (5%) Warfarin: 0 patients (0%)
Myocardial infarction	N/A	N/A	Dabigatran: 3 patients (2%) Warfarin: 0 patients (0%)
Dabigatran level	N/A	N/A	Dabigatran 86% time in therapeutic range vs. warfarin 49–51% time in therapeutic range
Conclusion	Lowest thrombi weight in the dabigatran group.	Mortality benefit of dabigatran over warfarin or no anticoagulation.	The use of dabigatran in patients with mechanical heart valves was associated with increased rates of thromboembolic and bleeding complications, as compared with warfarin.

* According to thromboembolic risk. ** Adjusted to obtain a trough plasma level of at least 50 ng per milliliter. SC: subcutaneously; PO: per os.; INR: international normalized ratio; BID: bidaily; N/A: not available.

**Table 2:** Rivaroxaban for mechanical valve anticoagulation.

Reference	L. E. Greiten et al. (2014) [21]	A. R. Durães et al. (2018) [4]	Roost et al. (2020) [22]	A. R. Durães et al. (2020) [23]
Setting	Swine heterotopic descending aorta valve conduit	Human-isolated mitral valve replacement > 3 months postoperatively	Human mechanical aortic valve replacement	Human aortic, mitral, or both valve replacement > 3 months postoperatively.
Treatment groups	10 swine: rivaroxaban 10 swine: enoxaparin 10 swine: no anticoagulation	7 patients No control group	10 patients No control group	23 rivaroxaban: 12 mitral, 6 aortic, 5 both 21 warfarin: 14 mitral, 2 aortic, 5 both
Valves used	St. Jude Masters Series (St. Jude Medical, Inc., St. Paul, MN, USA)	Not specified	Medtronic Open Pivot	Not specified
Dose	Enoxaparin 2 mg/kg BID SC Rivaroxaban 2 mg/kg BID PO	Rivaroxaban 15 mg BID PO	Rivaroxaban 20 mg OD PO	Rivaroxaban 15 mg BID PO Warfarin INR 2–3/2.5–3.5 * PO
Primary endpoint	Amount of valve thrombus at 30 days.	Adverse events of any kind at 3 months of follow-up.	Composite of major thromboembolic or bleeding events as well as death at 6 months of follow-up.	90 days of follow-up: Efficacy: composite of IS/TIA/SBI/SE Safety: major or clinically relevant non-major bleeding.
Results	No anticoagulation: thrombus 7/10 Enoxaparin: 8/10 Rivaroxaban: 4/10	No adverse events of any kind. No difference in echo graphic parameters. Eradication of spontaneous echo contrast in 2 patients	No adverse events of any kind. No difference in echo graphic parameters.	100% of follow-up and analysis in both groups. No statistical difference between rivaroxaban and warfarin in any outcome assessed. Numerically, rivaroxaban had a lower proportion of events.
Valve thrombus	No anticoagulation: 760 (0–2298) mg Enoxaparin: 717 (0–1490) mg Rivaroxaban: 210 (0–1337) mg	N/A	N/A	No signs of valve thrombosis or new intracardiac thrombus in either group.
Bleeding	None observed in either group	None observed	None observed	6 minor bleedings in each group
Stroke	N/A	None observed	None observed	Rivaroxaban: 1 TIA Warfarin: 1 SBI, 2 IS
Myocardial infarction	N/A	None observed	None observed	Warfarin: 1 acute myocardial infarction resulting in death.
Rivaroxaban level	Cmax: 65–231 µg/L Cmin: 3.89–95.5 µg/L	Not measured	Not measured	Not measured
Conclusion	Rivaroxaban significantly reduced thrombus weight and platelet deposition.	Use of rivaroxaban in patients with unstable INR after mitral valve replacement may be feasible.	Rivaroxaban treatment was safe, efficient, and feasible for the prevention of thromboembolic events in low-risk patients who received a mechanical aortic heart valve.	Rivaroxaban 15 mg BID had similar thromboembolic and bleeding events to warfarin in patients with mechanical heart valves.

SC: subcutaneously; PO: per os.; OD: once daily; BID: bidaily; N/A: not available; Cmax: maximum blood concentration; Cmin: minimum blood concentration; mg: milligram; µg/L: micrograms per liter; IS: ischemic stroke; TIA: transient ischemic attack; SBI: silent brain infarction; SE: systemic embolism; *: according to risk factors (left ventricle dysfunction, history of thromboembolism or atrial fibrillation).

**Table 3:** Apixaban for mechanical valve anticoagulation.

Reference	P. A. Lester et al. (2017) [6]	L. Van Hoof et al. (2022) [24]	PROACT-Xa (2023) [25]
Setting	Swine aortic heterotopic valve model	Swine mechanical valve in the pulmonary position.	Human aortic valve replacement >3 months postoperatively.
Treatment groups	5 swine: apixaban PO 4 swine: apixaban infusion 3 swine: warfarin PO 4 swine: no anticoagulation	2 swine: low dose 4 swine: intermediary dose 3 swine: high dose	420-patient apixaban cohort 414-patient warfarin cohort
Valves used	Not specified	21 mm On-X aortic valve	On-X aortic valve
Dose	Apixaban 1 mg/kg BID PO Apixaban 0.5 mg/kg bolus IV Warfarin 0.04–0.08 mg/kg (INR: 2–3) PO	Low dose: 5 mg BID for 10 weeks Intermediary dose: 5 mg BID for 6 weeks and then 10 mg BID for 4 weeks High dose: 15 mg BID for 10 weeks	Warfarin INR 2–3 PO Apixaban 5 mg BID *
Primary endpoint	Thrombus weight at 30 days for no anticoagulation and control groups. Thrombus weight at 14 days for apixaban infusion groups.	Thrombus presence and weight after 10 weeks.	Efficacy: valve thrombosis or valve-related thromboembolism Safety: major bleeding
Results	Apixaban PO: 3/5 thrombus Apixaban IV: 0/4 thrombus Warfarin PO: 2/3 thrombus	Low dose: 2/2 thrombus. Intermediate dose: 2/4 thrombus. High dose: 0/2 thrombus.	Terminated prematurely due to excess thromboembolic events in the apixaban cohort.
Valve thrombus	Apixaban PO: 357.5 ± 234.9 mg Apixaban IV: 61.1 ± 47.2 mg Warfarin 247.1 ± 134.3 mg No-anticoagulation: 1422 ± 676.4 mg	Low dose: 108.8 mg and 548.8 mg Intermediary dose: 65.5 mg and 41 mg. Two animals no thrombus. High dose: no thrombus.	Apixaban cohort: -3 valve thrombi -17 valve related thromboembolism -4.2%/patient-years Warfarin cohort: -0 valve thrombi -6 valve related thromboembolism -1.3%/patient-years
Bleeding	Apixaban PO: 0/5 Apixaban IV: 0/4 Warfarin: 2/3 Control group: 0/4	None observed	Major bleeding: -apixaban: 3.6%/patient-years -warfarin: 4.5%/patient-years
Stroke	None observed	None observed	Apixaban: 17 (2.9%/patient-years) Warfarin: 0 (0%/patients-years)
Myocardial infarction	None observed	None observed	Apixaban: 0 (0%/patient-years) Warfarin: 1 (0.2%/patients-years)
Apixaban level	Cmax: 214.67 ng/mL (SD: 91.51) AUC: 1390.00 ng∙h/mL (SD: 564.02)	Median apixaban peak plasma concentration Low dose: 43.6 ng/mL (range 23.1–64) Intermediary dose: 49.9 ng/mL (range 39.9–61) High dose: 49.9 ng/mL (range 44.6–82.9)	Not available for apixaban vs. warfarin; 72.7% time in the therapeutic range
Conclusion	Apixaban significantly reduced thrombus weight compared to no anticoagulation. Apixaban swine did not demonstrate bleeding events.	The results of this study suggest that even in highly thrombogenic situations, apixaban can effectively prevent thrombosis in MHVs. The study also found that increasing the dose of apixaban led to a decrease in thrombus formation, without increasing bleeding events.	Apixaban did not demonstrate noninferiority to warfarin and is less effective than warfarin for the prevention of valve thrombosis or thromboembolism in patients with an On-X mechanical aortic valve.

* Dose reduction to 2.5 mg BID when 2 of 3 are present: age > 80 years, weight 60 kg, creatinine > 1.5 mg/dL. BID: bidaily; IV: intravenously; PO: per os.; mg: milligram; Cmax: maximum blood concentration; AUC: area under the curve; ng/L: nanograms per liter.

## Data Availability

No new data were generated.
